# The (Bio)chemical Base of Flower Colour in *Bidens ferulifolia*

**DOI:** 10.3390/plants11101289

**Published:** 2022-05-11

**Authors:** Benjamin Walliser, Silvija Marinovic, Christoph Kornpointner, Christopher Schlosser, Mustafa Abouelnasr, Olly Sanny Hutabarat, Christian Haselmair-Gosch, Christian Molitor, Karl Stich, Heidi Halbwirth

**Affiliations:** 1 Institute of Chemical, Environmental and Bioscience Engineering, Technische Universität Wien, 1060 Vienna, Austria; benjamin.walliser@tuwien.ac.at (B.W.); silvija.miosic@tuwien.ac.at (S.M.); christoph.kornpointner@tuwien.ac.at (C.K.); christopher.schlosser@tuwien.ac.at (C.S.); abouelnasr.mustafa@gmail.com (M.A.); olly_hutabarat@yahoo.com (O.S.H.); christian.gosch@tuwien.ac.at (C.H.-G.); christian.molitor@tuwien.ac.at (C.M.); karl.stich@tuwien.ac.at (K.S.); 2 Department of Agricultural Technology, Hasanuddin University, Makassar 90245, Indonesia

**Keywords:** *Bidens ferulifolia*, Asteraceae, anthochlors, anthocyanins, okanin, carotenoid esters, lutein, yellow flower colour, UV-honey guides, flavonoid biosynthesis, chalcone 3′-hydroxylase

## Abstract

*Bidens ferulifolia* is a yellow flowering plant, originating from Mexico, which is increasingly popular as an ornamental plant. In the past few years, new colour combinations ranging from pure yellow over yellow-red, white-red, pure white and purple have emerged on the market. We analysed 16 *Bidens ferulifolia* genotypes to provide insight into the (bio)chemical base underlying the colour formation, which involves flavonoids, anthochlors and carotenoids. In all but purple and white genotypes, anthochlors were the prevalent pigments, primarily derivatives of okanin, a 6′-deoxychalcone carrying an unusual 2′3′4′-hydroxylation pattern in ring A. The presence of a cytochrome-P450-dependent monooxygenase introducing the additional hydroxyl group in position 3′ of both isoliquiritigenin and butein was demonstrated for the first time. All genotypes accumulate considerable amounts of the flavone luteolin. Red and purple genotypes additionally accumulate cyanidin-type anthocyanins. Acyanic genotypes lack flavanone 3-hydroxylase and/or dihydroflavonol 4-reductase activity, which creates a bottleneck in the anthocyanin pathway. The carotenoid spectrum was analysed in two *Bidens* genotypes and showed strong variation between the two cultivars. In comparison to anthochlors, carotenoids were present in much lower concentrations. Carotenoid monoesters, as well as diesters, were determined for the first time in *B. ferulifolia* flower extracts.

## 1. Introduction

*Bidens* L. is a globally spread genus belonging to Asteraceae [1], which is one of the largest and economically most important families of flowering plants [2]. Comprising more than 240 species [3], *Bidens* forms together with the closely related *Coreopsis* L. the two largest genera of tribe Coreopsidae [1]. *Bidens ferulifolia* (beggar tick, burr marigold) and hybrids thereof are the most commonly used *Bidens* species in horticulture [4].

*B. ferulifolia* is an upright growing bushy plant native to Mexico and Southern US [5], which has been used for almost three decades for growing in gardens, containers and mixed ornamental hanging baskets [4]. Despite being a perennial plant, it is typically discarded after the end of the season. Its heavy flower production makes it particularly attractive for pollinators. The first commercial *B. ferulifolia* cultivars were highly vigorous plants with limited market potential and exclusively yellow flowering. Since 2005, more compact cultivars were bred and in 2010, Florsaika from Japan obtained the first red and orange *B. triplinervia* through introgression [4,6]. *B. triplinervia* was crossable with *B. feru-lifolia*, resulting in F2 plants combining the coloration of *B. triplinervia* with the fitness of *B. ferulifolia*. The past five years, however, have seen an explosion in the number of commercial *Bidens* cultivars, extending the available colour spectrum from yellow to white, red and different types of bicoloured patterns [4]. While the pure white flowering cultivars are more popular in Northern Europe, the demand for orange and red flowering cultivars is rising in Southern Europe.

**Figure 1:**
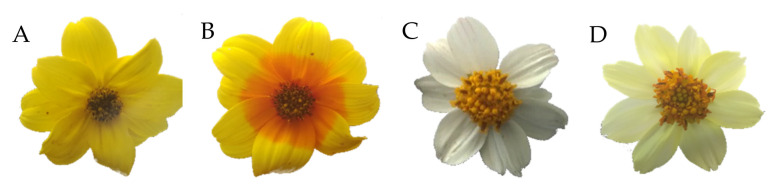
Effect of alkaline vapor on flower pigments. Left pair: Yellow *Bidens* cultivar accumulating carotenoids across whole petals and anthochlors in the basal parts of the petals, exemplified by cv. Mega Charm. Right pair: White *Bidens* cultivar accumulating flavones, exemplified by cv. Beedance White. (**A**) and (**C**) Native flowers. (**B**) After treatment with alkaline vapor, anthochlors in the basal part turn orange, while yellow carotenoids in the outer parts remain unaffected. (**D**) After treatment with alkaline vapor, flavones present in the petals turn a faint yellow colour.

The most common *B. ferulifolia* cultivars are pure yellow flowering as a result of the accumulation of two yellow pigments, the widespread carotenoids and the rare anthochlors (chalcones and aurones), which can be found only in a limited number of species [7]. Whilst carotenoids are distributed uniformly across the whole petal, anthochlors concentrate at the petal base and in the veins of the outer petal parts [8,9,10]. Due to the different UV absorbance of anthochlor pigments and carotenoids in combination with differences in the petal surface microarchitecture of the inner and outer parts (Schulte et al. 2019), this results in the presence of UV-honey guides attracting UV-sensitive insects as pollinators [11,12]. To the human eye, those patterns are not visible, and the petals appear monochromatic. Anthochlors can be, however, easily detected by exposing petals to ammonia or the alkaline vapor of a cigarette [13]. A bathochromatic shift of approximately 100 nm from the violet to the blue range of the spectrum is caused by the pH-dependent transition of the undissociated phenol groups to phenolates. The human eye perceives this shift of the wavelengths as a colour switch from yellow to red-orange [10] (Figure 1A,B). This is a rapid and anthochlor-specific detection method because carotenoids do not show such a colour shift. In the case of flavones and flavonols, which do not show absorption in the visible spectrum, only a faint yellow coloration may occur as a result of pH-dependent phenol dissociation (Figure 1C,D).

**Figure 2:**
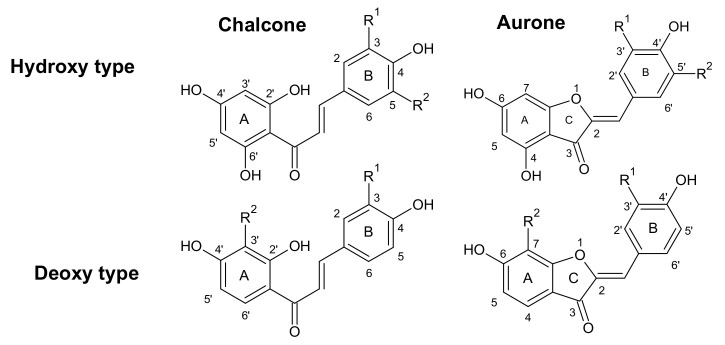
The general structure of anthochlors. Please note the divergent ring numbering in the chalcone and aurone structures. R^1^ = H or OH, R^2^ = H or OH.

The formation of anthochlors (chalcones and aurones) in *B. ferulifolia* has been studied earlier [10]. Anthochlors can be distinguished into two types, hydroxy and deoxy, defined by the presence or absence of a hydroxyl group at position 6′ of the A-ring (chalcones) or at position 4 of the B-ring (aurones) (Figure 2).

**Figure 3:**
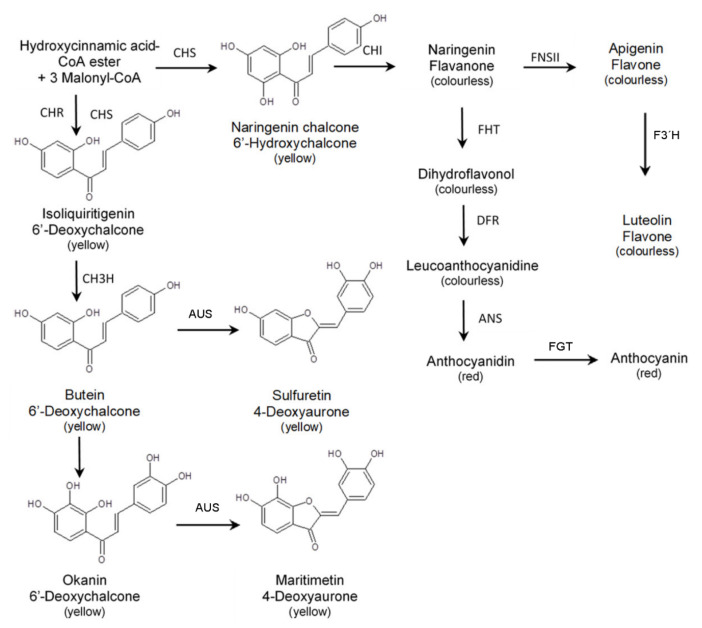
Overview of the biosynthetic pathway of flavonoids in *Bidens ferulifolia* and the general structure of chalcones and aurones. ANS: anthocyanidin synthase, AUS: aurone synthase, CH3H: chalcone 3-hydroxylase, CH3′H: chalcone 3′-hydroxylase, CHI: chalcone isomerase, CHR: chalcone reductase, CHS: chalcone synthase, DFR: dihydroflavonol 4-reductase, F3′H: flavonoid 3’-hydroxylase, FHT: flavanone 3-hydroxylase, FNSII: flavone synthase II.

Chalcones are formed by the enzyme chalcone synthase (CHS), which, acting alone, forms 6′-hydroxychalcones (Figure 3). These chalcones quickly isomerize either enzymatically or chemically to the corresponding colourless flavanones. The chemically stable 6’-deoxychalcones are formed by a mutual co-action of CHS and putative chalcone reductase (CHR) [14]. Accumulation of 6′-deoxychalcones is known to provide the base pigmentation for species of the Asteraceae family such as *Dahlia variabilis* [15]*, Cosmos sulphureus, Coreopsis grandiflora* or *B. ferulifolia* [16,17].

Chalcones are immediate precursors for the formation of aurones, which can therefore also be distinguished into hydroxy and deoxy types (Figure 3) [18].

Whilst the formation of 4-hydroxyaurones by the enzyme aureusidin synthase was intensively studied in *Antirrhinum majus* [19,20,21,22,23], the biosynthesis of 4-deoxyaurones was studied in different Asteraceae species, and addressed aurone formation [10,24,25] as well as the establishment of the B-ring hydroxylation pattern by an Asteraceae-specific chalcone 3-hydroxylase [26,27] or by aurone synthase (AUS) [28].

**Figure 4:**
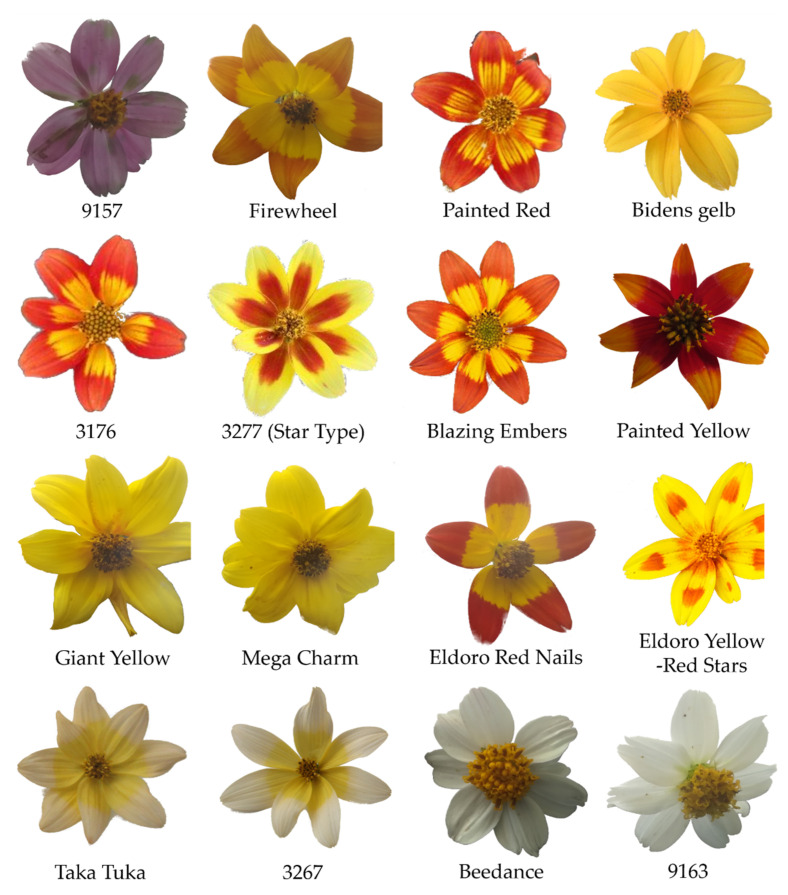
Overview of the *Bidens ferulifolia* genotypes analysed.

In this study, we investigated 16 *B. ferulifolia* genotypes (cultivars and breeding lines) exhibiting different colour combinations (Figure 4) to shed light on the underlying chemical and biochemical base of colour formation in this increasingly popular ornamental.

## 2. Results

### 2.1. Anthochlor and Flavonoid Pigment Composition

The pigment composition of the basal (base) and outer petal parts (tips) of the *Bidens ferulifolia* genotypes was analysed by HPLC after removal of glycoside moieties by acidic and enzymatic hydrolysis. Anthochlors, anthocyanidins and flavones were the predominantly present flavonoid pigments. In a few genotypes, low amounts of dihydroflavonols and flavonols were also found and are included in the column ‘Total’ (Table 1). The full spectrum present, including the individual compounds for each class, are provided in Supplementary Table S1. The pigment concentrations in the outer part were frequently lower than those in the basal parts.

Anthochlors were present in all *Bidens* genotypes with the exception of the faint purple line 9157 and the pure white genotypes, cv. Beedance White and line 9163, in which no anthochlor pigments were found. Okanin was the most abundant pigment within the anthochlors, reaching up to 11 mg/g FW in some of the genotypes, whereas the concentrations of butein and maritimetin were below 1 mg/g FW. Flavones were found in all but one genotype. In the majority, only luteolin, but no apigenin could be detected (Supplementary Table S1). In comparison to anthochlors, flavone concentrations were much lower, reaching a maximum of 2.9 mg/g FW in the pure white and the faint purple genotypes. In these, however, small amounts of apigenin were also present, in addition to luteolin. In yellow and red *Bidens* petals, luteolin concentrations were lower, ranging from 0.06–1 mg/g FW. Red petal parts also accumulated anthocyanins, particularly cyanidins, and in one case also traces of peonidin (Supplementary Table S1). The concentrations of anthocyanins were comparable to those of flavones and, thus, much lower than the anthochlor concentrations. The highest anthocyanin concentrations were observed in the red outer parts of cv. Firewheel, cv. Eldoro Red Nails and cv. Painted Red. In red-yellow flowering genotypes, a sharp separation of yellow and red tissues was not always possible, as the areas were often intermingled (Figure 4). Of the class of dihydroflavonols, dihydroquercetin (DHQ) was almost exclusively present, but only in the faint lilac and the white genotypes and in some of the red tissues. The highest concentrations were found in the faint purple line 9157, where small amounts of dihydrokaempferol (DHK) in addition to DHQ were also present.

### 2.2. Enzyme Activities of the Flavonoid and Anthochlor Pathway

The activities of selected enzymes involved in flavonoid biosynthesis were measured. An overview is provided in Table 2. All genotypes showed comparable CHS activity regardless of the flower colour, though the CHS activity was two to three times higher in monochromatic white and purple genotypes compared with the yellow and yellow-red cultivars. A lack or low activity of flavanone 3-hydroxylase (FHT) and/or dihydroflavonol 4-reductase (DFR) was found in all cultivars and tissues, respectively, in which no anthocyanins were formed (Table 2). Both enzymes were affected in the yellow cv. Mega Charm, the yellow edge of cv. Eldoro Yellow Red Star, in the yellow parts of the cultivars Blazing Embers and Eldoro Red Nails and in the cream parts of cv. Taka Tuka and line 3267. DFR activity, but no FHT activity, was detected in the red-yellow parts of cv. Painted Red, the yellow edge of line 3277, the yellow tips of cv. Painted Yellow and the yellow cv. Giant. In all other acyanic genotypes, the absence of DFR provided a sufficient explanation for the lack of anthocyanins. Flavone synthase II (FNSII) activity could be detected in enzyme preparations from all lines and showed the highest activity in the red-yellow cultivars. CH3 H and CH3′H could also be detected in all genotypes.

**Figure 5:**
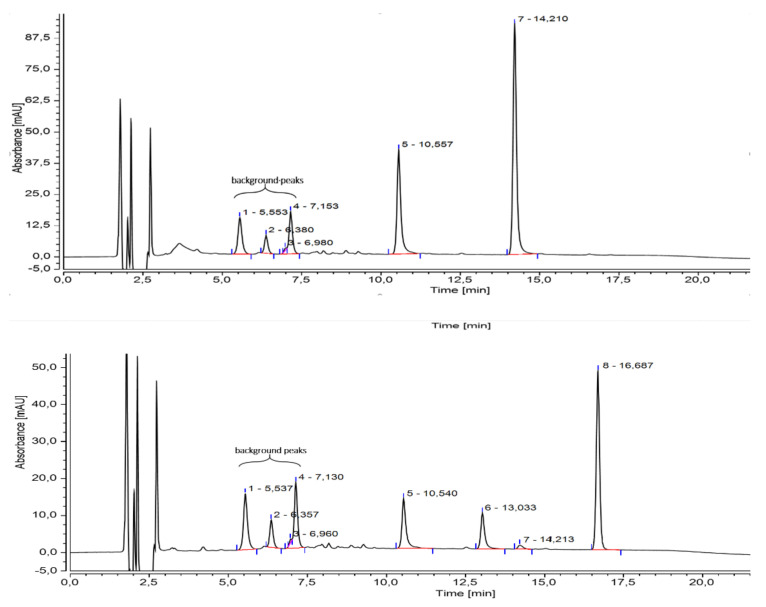
HPLC chromatograms at 385 nm from incubation of enzyme preparations from *Bidens* petals in the presence of NADPH using butein (top) and isoliquiritigenin (bottom) as substrates. Peaks 1–4 are background peaks resulting from remnants of anthochlor and flavonoid glycosides left by the enzyme preparation. 5: okanin, 6: 3′-hydroxyisoliquiritigenin, 7: butein, 8: isoliquiritigenin. Please note: the numbers after the retention labels 1-8 are the retention times in seconds, with a decimal comma instead of a period.

In the frame of our investigations, chalcone 3′-hydroxylase activity was demonstrated for the first time. Incubation of butein with enzyme preparations from *Bidens* petals in the presence of NADPH led to the formation of okanin, which was identified by comparison with the authentic reference compound by HPLC and LC-MS (Figure 5, top). When isoliquiritigenin was used as a substrate, simultaneous activities of CH3H and CH3′H were observed, resulting in the formation of butein, 3′-hydroxyisoliquiritigenin and okanin (Figure 5, bottom). All products were identified by LC-MS (Supplementary Table S3).

### 2.3. Carotenoids

**Figure 6:**
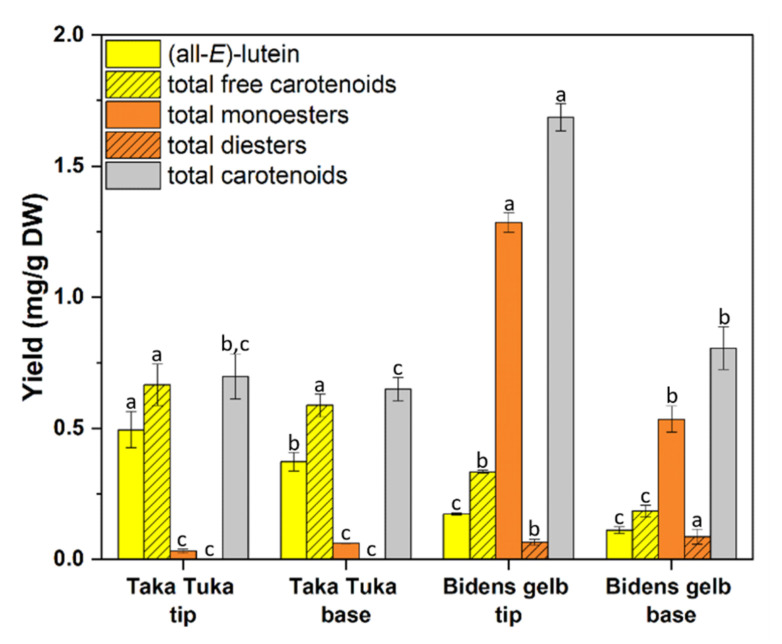
Carotenoid yields (mg/g DW) in cultivars Taka Tuka and Bidens gelb (*n* = 3, SD). The same letter (a–c) indicates no statistically significant difference across species for the same carotenoid according to Duncan (*p* < 0.05).

Apart from flavonoids and anthochlors, the carotenoids present in *B. ferulifolia* flowers were analysed in two selected cultivars, the uniformly yellow flowering cv. Bidens gelb and the yellow cream cv. Taka Tuka. As for flavonoids and anthochlors, basal (base) and outer parts (tips) of the petals were analysed separately. The concentrations and compositions of carotenoids, which were extracted after freeze drying, varied strongly between the two analysed cultivars. Cv. Bidens gelb mainly accumulated esterized carotenoids, whereas in cv. Taka Tuka more than 90% free carotenoids were found (Figure 6 and Supplementary Table S4). Examples for HPLC chromatograms and an overview of the structures identified in the *B. ferulifolia* petals is provided in Supplementary Figures S1 and S2.

In both cultivars, the predominant xanthophyll was (all-*E*)-lutein, reaching up to 0.50 mg/g DW in cv. Taka Tuka (Figure 6, Supplementary Tables S4 and S5). In addition, high yields of the isomer (13*Z*)-lutein were found (up to 0.11 mg/g DW) in the extracts of cv. Bidens gelb. Thus, the total free carotenoid content consisted of 65% (base) and 75% (tip) lutein isomers in cv. Taka Tuka and 83% (base) and 84% (tip) in cv. Bidens gelb. In addition, further minor xanthophylls were determined in both cultivars, namely (all-*E*)-zeaxanthin, (all-*E*)-violaxanthin and (all-*E*)-antheraxanthin, whereas (all-*E*)-β-carotene was only found in cv. Taka Tuka. Concentrations were, however, only in the range of µg/g DW.

Some of the carotenoids were present as monoesters and diesters (Figure 6, Supplementary Table S5). In cv. Bidens gelb, up to 1.29 mg/g DW of carotenoid monoesters were found, predominantly lutein palmitates (base: 45%; tip: 52%) and lutein myristates (base: 28%; tip: 25%), but also smaller amounts of violaxanthin monoesters (base: 8%; tip: 6%) (Figure 6 and Supplementary Table S4). The total diester yields were 87 µg/g DW (base) and 66 µg/g DW (tip), consisting mainly of lutein diesters (base: 73%; tip: 68%) and violaxanthin diesters (base: 27%; tip: 32%) (Figure 6 and Supplementary Table S4). In cv. Taka Tuka, in contrast, no diesters could be detected. Carotenoid monoesters reached only a maximum of 59 µg/g DW in this cultivar and were predominantly lutein myristate (base: 30%; tip: 38%) esters and lutein palmitate esters (base: 65%; tip: 60%).

In cv. Taka Tuka, no significant differences were found between the bases and tips of the petals; 0.70 mg/g DW of total carotenoid in the tips and 0.65 mg/g dray weight in the base were yielded. The basal and outer flower parts seem to accumulate comparable amounts of free and bound carotenoids and were dominated by free carotenoids (Figure 6, Supplementary Table S4). In cv. Bidens gelb, in contrast, significantly higher total carotenoid contents of 1.69 mg/g DW in the tips and 0.81 mg/g DW in the base were found. The relative contents of free carotenoids and carotenoid esters were, however, comparable. For both parts, the total carotenoid content consists of approx. 20% free carotenoids and 80% carotenoid esters.

## 3. Discussion

In *B. ferulifolia*, flower colour is determined by four main pigment classes, anthochlors, flavones, anthocyanins and carotenoids. The biosynthetic pathway of the first three shares common intermediates, and therefore, the formation of anthocyanins, anthochlors and flavones is competitive. As well as contributing to flower coloration, anthochlors are also responsible for the formation of UV-honey guides, making *Bidens* a model plant for the study of anthochlor-based UV-honey guides. The biosynthesis of anthochlors and antho-cyanins starts both from *p-*coumaroyl-CoA and involves CHS (Figure 3). The simultaneous action of CHR initiates the formation of anthochlors of the deoxy type, which is found in some members of the Asteraceae family. The pathway leading to flavones and anthocyanins diverges downstream at the level of flavanones (Figure 3). The flux into the individual pathways depends not only on the presence or absence of enzymes but also of relative activities of enzymes competing for the same substrate.

In this study, we measured the activities of selected enzymes involved in flavonoid and anthochlor formation in order to elucidate potential bottlenecks in the pathway. CHS as the key enzyme of the flavonoid pathway is essential for the formation of all three pigment types (Figure 3). FHT and/or DFR are further important enzymes for the formation of anthocyanins, whereas FNSII is the key enzyme for flavone formation and competing with FHT for the same substrate. CH3H and CH3′H are part of the anthochlor pathway determining the hydroxylation pattern of the prevalently present anthochlor okanin. Anthocyanidin synthase (ANS), which is the last enzyme in anthocyanidin formation, and chalcone reductase (CHR), which interacts with CHS to start the 6′-deoxychalcone formation, were not included, as no reliable assays for their activities are available so far.

### 3.1. Anthocyanins

Whilst in the early yellow cultivars, carotenoids, anthochlors and flavones were exclusively present, anthocyanins have entered the scene with the introduction of red flowering cultivars. Meanwhile a large number of anthocyanin-accumulating cultivars exist. Most of them, however, have bicolored flowers, where anthocyanins are located in the outer parts of the flower and predominantly in the adaxial epidermis, although cultivars exist where anthocyanins are concentrated at the base or appear as red stars on a yellow background. In some cultivars, there is a sharp clear border between red and yellow sections (e.g., Eldoro Red Nails, Beedance, Firewheel), but in many cultivars the borders are blurred (Figure 4). In addition, the intensity and distribution of the red colour of a cultivar depends on the cultivation and specifically requires high light intensities [29,30]. The importance of light for the induction of anthocyanin formation has been previously reported [31].

From a biochemical point of view, the formation of anthocyanins requires the presence of the full set of enzyme activities of the pathway to form the basic anthocyanidin structure. In all genotypes, except line 3176, the absence of anthocyanins was clearly reflected by a lack of the activities of one or both of the enzymes FHT and DFR, with relatively balanced distribution of lines in which DFR or FHT was affected. In the yellow base of line 3176 the absence of anthocyanins could not be completely explained, because apart from FHT activity, DFR could also be detected, albeit only in low activities. In this case, a lack of ANS activity could additionally contribute. In the white genotypes, the absence of anthocyanins is based on the lack of DFR activity, which creates a bottleneck resulting in the accumulation of flavones and dihydroflavonols, which was rarely observed in the analysed genotypes. In the yellow-cream genotypes, DFR activity was generally missing, but in the cream-colored outer parts, FHT was affected as well.

Interestingly, high DFR activity comparable to the yellow-red genotypes was measured in the monochromatically yellow cv. Giant Yellow. Such a genotype carries a high potential for breeding new lines, especially in combination with the yellow-cream and pure white DFR-lacking genotypes, potentially leading to new and striking colour combinations, such as red-white or pure, intense red, by reinstalling the pathway to anthocyanins.

The highest DFR activity was detected in the faint purple line 9157, which strongly contrasted with the highest concentrations of dihydroflavonols found in this genotype (Supplementary Table S1). This points at a bottleneck located downstream of DFR, creating a tailback of dihydroflavonol intermediates, which is in line with the low anthocyanin concentrations resulting in the faint purple colour.

### 3.2. Anthochlors

In the yellow-red and yellow flowering genotypes, anthochlor pigments were found in the basal and outer parts of the flowers. In the outer parts, however, concentrations were much lower,and were restricted to the veins as reported earlier [24], which is also exemplarily shown for cvs. Giant Yellow, Mega Charm and Taka Tuka in Supplementary Figure S3. This is also particularly relevant in the yellow cream genotypes, in which the outer cream parts show significantly lower concentrations of anthochlors and flavones than the yellow basal parts, as expected. However, in comparison with pure white genotypes, which completely lack anthochlors, relatively high concentrations of anthochlors are found in the cream parts. Due to the presence exclusively in the veins (Supplementary Figure S3), the outer part of these varieties appear cream. The absence of anthochlors in the pure white and the faint purple genotypes points at the absence of CHR activity, which cannot be proven, as the formation of 6′-deoxychalcones in the Asteraceae species is not sufficiently understood so far [32].

In contrast to earlier studies [10], we found in the current genotypes primarily chalcones and only low amounts of aurones (Supplementary Table S1). Okanin was prevalently present, a 6′-deoxychalcone exhibiting a rare hydroxylation pattern in the A-ring, because in addition to the commonly found hydroxy groups in position 2′ and 4′, a hydroxy group in position 3′ is present. This corresponds to an additional hydroxy group in position 8 of 5-deoxyflavonoids. In the case of the common 6′-hydroxychalcones, position 3′ corresponds to both positions 6 and 8 of 5-hydroxyflavonoids, respectively, because of the free rotation of ring A. Flavonoids carrying additional hydroxy groups in the A-ring are rare, but have been reported for a few Asteraceae species, e.g., gossypetin (8-hydroxyquercetin) in *Chrysanthemum segetum* and *Gossypium hirsutum* [13,33]. Cytochrome-P450-dependent monooxygenases are frequently responsible, but also FAD dependent enzymes [34]. Whereas the establishment of the B-ring hydroxylation pattern of chalcones is well investigated and involves a cytochrome P450 dependent monooxygenase [26], the introduction of a hydroxy group in position 3′ has not been investigated so far. Using enzyme preparations of *Bidens* petals, we report here for the first time the presence of an enzyme catalysing the introduction of a hydroxyl group in position 3′ of 6′-deoxychalcones. The reaction was dependent on NADPH, and conversion rates were not further increased by FAD. Both isoliquiritigenin and butein are accepted as substrates, but in the case of isoliquiritigenin, the reaction cannot be observed independently from the CH3H reaction, which is also present in the enzyme preparation and also uses NADPH as a cofactor. In the presence of the cytochrome-P450-specific inhibitors ketoconazole and tetcyclacis, the hydroxylation rate in position 3′ decreased by 90%, thereby pointing at the involvement of a cytochrome-P450-dependent monooxygenase.

### 3.3. Carotenoids

Carotenoids have been reported in various Asteraceae species [35]; however, for *B. ferulifolia* little is known. Valadon et al. [36] carried out a study to determine carotenoids in *B. ferulifolia* flowers and described the presence of carotenes (α, β) and epoxycarotenes, as well as three xanthophylls, namely lutein, auroxanthin and flavoxanthin. However, until now, *B. ferulifolia* extracts have not been analysed by modern analytical techniques. For this purpose, the flower petals were freeze dried to improve the extractability of carotenoids by the removal of water, and to enhance the ratio of the amount of carotenoids to the initial plant weight. Thus, carotenoid concentrations are provided on a DW base and can, therefore, not be directly compared with flavonoid and anthochlor concentrations in Table 1. It seems clear, however, that in comparison with anthochlors, carotenoids are the minor yellow pigments in *Bidens* petals.

Mass spectrometric analysis led to the identification of lutein and β-carotene, which have both been found before in *B. ferulifolia* extracts [36]. In addition, various xanthophylls were identified for the first time, namely violaxanthin, luteoxanthin, antheraxanthin, lutein 5,6-epoxide and zeaxanthin (Supplementary Table S5). Those carotenoids have been described in other yellow flowering Asteraceae species before and are known as part of the carotenoid biosynthesis [35]. For some carotenoids, e.g., lutein or violaxanthin, among others, several *cis* isomers were determined, which can be the result of light exposure, heat or acids [37].

Apart from that, carotenoid monoesters, as well as diesters, were determined for the first time in *B. ferulifolia* flower extracts. Mainly, myristate and palmitate esters of lutein or violaxanthin were characterized (Supplementary Table S4). Several identified xanthophyll esters have been shown to be accumulated in flowers of marigold (*Tagetes* sp.) [38].

In addition, a greater variety of carotenoid esters was found in cv. Bidens gelb when compared with cv. Taka Tuka, where mainly lutein esters were characterized (Figure 6, Supplementary Tables S4 and S5). It can be noted that xanthophyll diesters were solely determined in cv. Bidens gelb, whereas β-carotene was only found in cv. Taka Tuka. Hence, the content of carotenoids strongly depends on the genotype.

## 4. Materials and Methods

### 4.1. Plant Material

The investigations were carried out on 16 *Bidens ferulifolia* L. genotypes (cultivars and lines) which are listed in Figure 4 and Table 1 and Table 2. Petals of the cultivars Firewheel (Florsaika, Netherlands), Painted Red (Beedance Painted Red, MNP Suntory, Netherlands), Eldoro Red, Eldoro Yellow, Giant Yellow, Megacharm (Danziger, Israel) and Beedance White (MNP Suntory, Netherlands) were harvested from the collection of Selecta One GmbH, Stuttgart (Germany); samples of the cultivars 9163, 9177 and Taka Tuka were obtained from Volmary GmbH, Münster (Germany). Petals from fully developed flowers were harvested and separated into the base (lower 40%) and the tip (upper 40%). The intermediate area (central 20%) was discarded in order to avoid cross contamination. The separated parts were shock frozen in liquid nitrogen and stored at −80 °C.

### 4.2. Chemicals

Isoliquiritigenin, butein, apigenin, luteolin, naringenin, eriodictyol, dihydrokaempferol, dihydroquercetin, marein, maritimein, peonidin and cyanidin were purchased from Extrasynthese (Genay, France). Okanin and maritimetin were obtained from marein and maritimein by enzymatic hydrolysis, as described previously [32]. Lutein (≥95%) and β-carotene (≥98%) were obtained from Extrasynthese (Genace, France). For HPLC mobile phases, acetonitrile hypergrade LC-MS (and formic acid 98–100% and ultra-pure water (Type 1, Direct-Q^®^ 3UV)) were purchased from Merck (Darmstadt, Germany). NADPH was purchased from LabConsulting (Vienna, Austria).

### 4.3. Anthochlor and Flavonoid Analysis

Analysis for flavonoid class and anthochlors was conducted according to [39]. An amount of 0.5 g (fresh weight) of frozen petal material was macerated in a mortar with 1 mL 2 M hydrochloric acid in methanol and extracted for 5 min at room temperature. After centrifugation, 40 µL supernatant was incubated with 160 µL 4 M HCl for 60 min at 95 °C for anthocyanin analysis. For enzymatic hydrolysis of flavones, flavonols and anthochlors, 20 µL of the supernatant was subjected to enzymatic hydrolysis by 10 U Naringinase (Sigma-Aldrich, Vienna, Austria) and incubated for 20 min at 40 °C in 0.1 M McIlvaine buffer pH 4.0. The reaction was stopped by addition of 30 µL methanol and used in HPLC analysis according to [40]. A Thermo Scientific Dionex UltiMate(R) 3000 RSLC System with DAD-3000RS Photodiode Array Detector (Thermo Scientific, Vienna, Austria) using an Acclaim™ column RSLC 120 C18, 2.2 µm, 120 Å, 2.1 mm × 150 mm (Dionex bonded Silica Products: No. 071399) was operated at 25 °C. For analysis of flavones, flavonols and anthochlor pigments, elution solvents were (A) 0.1% formic acid in water and (B) 0.1% formic acid in acetonitrile (gradient: 3 min pre-run at 20% B, 0–15 min 20–53% B, 15–20 min 53–95% B, 20–31 min 95% B, 31–35 min 20% B, flow rate 0.2 mL/min). The compounds were detected at 385 nm and identified by retention times and comparison of the UV-VIS spectra from 190 to 800 nm using authentic reference compounds. The concentrations were calculated from the peak areas of samples and standard lines obtained with the respective reference compounds.

### 4.4. Carotenoid Analysis

For carotenoid analysis, part of the separated flower petals of the cultivars Taka Tuka and Bidens gelb were freeze dried until weight consistency. Subsequently, ultrasound assisted extraction (UAE) with a Bandelin SONOREX RK 100 SH (35 KHz, 100 W, VWR, Vienna, Austria) was performed at room temperature for 2 h. Thereafter, 0.05–0.14 g dried and ground flower powder was extracted with 0.9–1.4 mL MeOH. The samples were centrifuged, filtered through a 0.22 µm filter and stored at −80 °C under dark conditions until analysis.

Identification of carotenoids was performed by UHPLC-DAD-APCI-MS. The methanolic extracts were injected to a 1290 Infinity LC System (Agilent Technologies, USA) equipped with an Acclaim C30 column (3 μm, 2.1 mm × 100 mm, Dionex bonded silica products: NO. 01834074, Thermo Fisher Scientific, Vienna, Austria) coupled to a G7117C diode array detector (450 nm) and subsequently followed by a 6545 LC/Q-TOF (Agilent Technologies, Santa Clara, USA) with a multimode ion source operating in APCI positive mode (range: 100–1700 *m/z*, scan rate: 2 spectra/sec). A combination of buffer A: 91% MeOH, 5% methyl *tert*-butyl ether (MTBE), 3.9% H_2_O and 0.1% formic acid; and buffer B: 46% MeOH, 50% MTBE, 3.9% H_2_O and 0.1% formic acid was used as mobile phase. Following gradient program was applied: 3 min pre-run at 1% B, 0–5.5 min at 1% B, 5.5–19 min to 1–75% B, 19–20 min to 75–100% B, 20–30 min 100% B, 30–31 min 1% B, 31–34 min 1% B, flow 10 min at 95 vol% (B), 1 min to 20 vol% (B) and post-run 10 min 20 vol% (B). A solvent cut was performed for 0.5 min. The flow rate was set to 0.35 mL/min and the column oven was operated at 18 °C. The recently described method [41] was adapted and modified, and identification of carotenoids was performed based on their fragmentation, retention time and the substance-specific absorbance [38,42,43,44,45]. A list of all identified carotenoids for both cultivars is presented in the Supplement Materials (Supplementary Table S4).

Quantification of carotenoids was performed by the same HPLC system as described in Section 4.3. The same C30 column and separation parameters as previously described for UHPLC-DAD-APCI-MS were carried out. External calibration was performed with lutein and β-carotene. Additional identified xanthophylls and xanthophyll esters are expressed as lutein equivalents. Results are expressed as mg per gram dried flower petals (mg/g DW).

### 4.5. Enzyme Assays

Assays for CHS, CH3H, FNSII, DFR and FHT were performed as described earlier [10]. For CH3′H assays, the reaction contained in the final volume of 100 µL: 40 µL enzyme preparation from petals, 10 µM isoliquiritigenin or butein, 0.05 nM NADPH and 55 µL 0.1 M KH_2_PO_4_/K_2_PO_4_ buffer pH 7.5 containing 0.4% Na ascorbate. The reaction mixture was incubated at 30 °C for 30 min and stopped with 20 µL of 20% acetic acid in acetonitrile. After centrifugation at 16,000 × *g* for 5 min the reaction solution was filtered through a 0.22 µm PTFE membrane. The CH3′H substrates and reaction products isoliquiritigenin, butein, 3′-hydroxyisoliquiritigenin and okanin were identified by high-performance liquid chromatography as described in Section 4.2. For product identification, liquid chromato-graphy coupled to mass spectrometry (HPLC-MS-MS) was performed. An amount of 6 µL of the assay was injected on a 1290 Infinity II LC System (Agilent Technologies, Santa Clara, CA, USA) equipped with a 1260 Infinity II diode array detector (DAD). The separation was performed on a ZORBAX Eclipse Plus C18 Rapid Resolution 1.8 µm, 2.1 mm × 150 mm column (Agilent, Santa Clara, USA) at 35 °C and 0.3 mL/min. The mobile phases were (A) H_2_O with 0.1% formic acid and (B) acetonitrile with 0.1% formic acid. The gradient was set as follows: 0−5 min, 5−15% B; 5−11 min, 15−53% B; 11−15 min, 53−100% B; 21.5 min, 100% B; 21–22 min 100–5% B, 22–26 min 5% B.

Mass detection was performed using an Agilent High-Resolution-y MS 6545 Q-TOF with a Multimode Ion Source (Agilent Technologies, Santa Clara, USA). The main instrumental conditions were as follows: negative electrospray ionization mode, MS scan range was from *m*/*z* 100 to 1000, product ion scan range from *m*/*z* 50 to 300, capillary voltage 2.0 kV; gas temperature 350 °C; vaporizer temperature 220 °C; gas flow 5 L/min; nebulizer 60 psi; fragmentor voltage 180 V; skimmer 75 V. Nitrogen was used as nebulizer and auxiliary gas. The collision energy for the fragmentation was set on 15 eV.

Data acquisition was carried out using Agilent Mass Hunter Workstation Data Acquisition (Agilent Technologies, Santa Clara, CA, USA) and evaluated using Agilent MassHunter Qualitative Analysis 10.0. Identifications were based on chromatographic elution time, accurate Mass, MS/MS fragmentation pattern, and comparisons with available standards.

### 4.6. Statistical Analysis

The statistical analysis of HPLC and enzyme activity assays was performed using RStudio2022.02.0 + 443 and R v 4.1.2 with the library package “agricolae” [46]. Group-wise comparison of enzyme activity or flavonoid contents between different genotypes was calculated using Duncan’s new multiple range test (MRT) [47]. The significance level was set to α = 0,05 (5%).

## 5. Conclusions

*B. ferulifolia* exhibiting other colours than yellow are relatively new to the market and the biochemical base behind the coloration has not been given a great deal of attention. In this study, we took a closer look at differently coloured *B. ferulifolia* cultivars ranging from pure yellow over red-yellow to white-yellow bicolored and pure white cultivars. To our knowledge, this is the first study dealing with these colour combinations in *B. ferulifolia*.

One of the goals of this study was the search for candidates for breeding new colour combinations. Potential breeding partners are the yellow-red cv. Painted Red, the pure yellow cv. Giant Yellow and the pure white genotypes. Whilst the first two genotypes possess a functional DFR, a lack of FHT activity prevents the synthesis of anthocyanins and vice versa in the pure white genotypes. Introducing a functional enzyme may lead to new colour combinations such as red-white or pure red. Follow-up studies with these genotypes may include investigations of the gene level such as expression studies and sequencing. Nonetheless, this study shows that biochemistry can provide useful information for plant breeders.

## Figures and Tables

**Table 1:** Content of anthochlors and flavonoids (**µg/g fresh weight)**, quantified as aglycones after acidic hydrolysis, in *B. ferulifolia* cultivars. Subtotals for anthochlors and each flavonoid class are shown. The column ‘Total’ includes anthochlors, flavones, anthocyanins and dihydroflavonols. Qualitative and quantitative composition within classes is shown in Supplementary Table S1. Same letters (a–m) indicate no statistically significant differences according to Duncan (*p* < 0.05) between different genotypes, n.d.: not detected.

Cv./Line	Petal Part	Colour	Anthochlors	Flavones	Anthocyanins	Total
			µg/g fresh weight			
9157	entirety	Purple	n.d.^l^	2944^b^	345^d^	4632
Firewheel	base	Red	6184^efgh^	787^fg^	249^e^	7381
	tip	Red	5272^ghi^	1090^e^	1806^a^	8753
Painted Red	base	Red- Yellow	9223^abcd^	594^hi^	19^f^	9841
	tip	Red	8334^bcde^	559^hi^	1597^b^	10560
Bidens gelb	entirety	Yellow	1987^kl^	152^lm^	n.d.^f^	2139
3176	base	Yellow	3556^ijk^	338^jk^	n.d.^f^	3894
	tip	Red	11095^a^	1287^d^	n.d.^f^	12382
3277 (Star type)	edge	Yellow	6269^efgh^	193^kl^	n.d.^f^	6461
	star	Red	8315^bcde^	794^fg^	n.d.^f^	9109
Blazing Embers	base	Yellow	7202^cdefg^	685^gh^	n.d.^f^	7886
	tip	Red	3253^ijk^	176^klm^	n.d.^f^	3428
Painted Yellow	base	Red	2015^kl^	n.d.^m^	517^c^	2717
	tip	Yellow	1577^kl^	n.d.^m^	250^e^	1843
Giant	base	Yellow	8110^bcdef^	533^hi^	5^f^	8651
	tip	Yellow	1593^kl^	39^lm^	28^f^	1665
Mega Charm	base	Yellow	6829^defgh^	764^fg^	n.d.^f^	7593
	tip	Yellow	2003^kl^	170^lm^	n.d.^f^	2173
Eldoro Red Nails	base	Yellow	4514^hij^	458^ij^	n.d.^f^	4972
	tip	Red	4788^ghij^	421^ij^	1627^b^	7300
Eldoro Y. Red Star	star	Red	10000^ab^	583^hi^	353^d^	10966
	edge	Yellow	1545^kl^	46^lm^	n.d.^f^	1591
Taka Tuka	base	Yellow	9513^abc^	901^f^	n.d.^f^	35998
	tip	Cream	2543^jkl^	112^lm^	n.d.^f^	11088
3267	base	Yellow	9291^abcd^	1371^d^	n.d.^f^	41143
	tip	Cream	5627^fghi^	416^ij^	n.d.^f^	18807
Beedance White	entirety	White	n.d. ^l^	3252^a^	7^f^	3484
9163	entirety	White	n.d. ^l^	1806^c^	n.d.^f^	1852

**Table 2:** Selected enzyme activities of the flavonoid and anthochlor pathways detected from enzyme preparations of *B. ferulifolia* cultivars. Same letters (a–i) in a column indicate no statistically significant differences according to Duncan (*p* < 0.05) between different genotypes, n.d.: not detected. An extended version was included as Supplementary Table S2.

Cv./Line	Petal	Colour	CHS	FHT	DFR	FNSII	CH3H	CH3′H
	Section		Specific Activity (nmols^−1^kg^−1^)					
9157	entirety	Purple	852 ^bcd^	321 ^b^	1286 ^a^	183 ^de^	2121 ^a^	1642 ^ghi^
Firewheel	base	Red	383 ^ghi^	90 ^ef^	473 ^cd^	417 ^ab^	3663 ^a^	7472 ^a^
	tip	Red	253 ^i^	145 ^de^	892 ^b^	469 ^a^	4463 ^a^	3367 ^defgh^
Painted Red	base	Red- Yellow	774 ^cde^	n.d. ^g^	605 ^c^	435 ^a^	3568 ^a^	4701 ^bcd^
	tip	Red	658 ^cdefg^	546 ^a^	975 ^b^	391 ^abc^	3704 ^a^	5989 ^ab^
Bidens gelb	entirety	Yellow	465 ^fghi^	69 ^fg^	n.d. ^h^	162 ^de^	2988 ^a^	3414 ^defgh^
3176	base	Yellow	219 ^i^	219 ^c^	11 ^h^	400 ^abc^	3519 ^a^	4000 ^cdef^
	tip	Red	250 ^i^	249 ^c^	188 ^fg^	418 ^ab^	5589 ^a^	3980 ^cdef^
3277 (Star type)	edge	Yellow	489 ^efghi^	n.d. ^g^	55 ^gh^	383 ^abc^	4386 ^a^	4762 ^bcd^
	star	Red	734 ^cdef^	381 ^b^	240 ^ef^	315 ^abcd^	3781 ^a^	5619 ^bc^
Blazing Embers	base	Yellow	226 ^i^	n.d. ^g^	20 ^g^	118 ^de^	2310 ^a^	3159 ^defghi^
	tip	Red	276 ^hi^	242 ^c^	153 ^fgh^	179 ^de^	1996 ^a^	2184 ^fghi^
Painted Yellow	base	Red	446 ^fghi^	180 ^cd^	377 ^de^	231 ^bcde^	2619 ^a^	2454 ^efghi^
	tip	Yellow	483 ^cde^	n.d. ^g^	18 ^h^	131 ^de^	2870 ^a^	3101 ^cdef^
Giant	base	Yellow	480 ^fghi^	n.d. ^g^	255 ^ef^	172 ^de^	3640 ^a^	3958 ^cdef^
	tip	Yellow	403 ^ghi^	n.d. ^g^	559 ^c^	104 ^e^	1769 ^a^	3505 ^defgh^
Mega Charm	base	Yellow	430 ^ghi^	n.d. ^g^	n.d. ^h^	46 ^e^	2795 ^a^	4135 ^cde^
	tip	Yellow	217 ^i^	n.d. ^g^	n.d. ^h^	70 ^e^	1730 ^a^	1366 ^i^
Eldoro Red Nails	base	Yellow	412 ^ghi^	n.d. ^g^	n.d. ^h^	218 ^cde^	3880 ^a^	3126 ^defghi^
	tip	Red	436 ^ghi^	375 ^b^	1028 ^b^	221 ^cde^	3617 ^a^	3400 ^defgh^
Eldoro Yellow Red Star	star	Red	561 ^efghi^	187 ^cd^	435 ^d^	184 ^de^	2878 ^a^	2437 ^efghi^
	edge	Yellow	490 ^defgh^	n.d. ^g^	n.d. ^h^	45 ^e^	1363 ^a^	1751 ^ghi^
Taka Tuka	base	Yellow	827 ^bcd^	30 ^fg^	n.d. ^h^	137 ^de^	2683 ^a^	2736 ^efghi^
	tip	Cream	786 ^bcd^	n.d. ^g^	n.d. ^h^	60 ^e^	1732 ^a^	3376 ^defgh^
3267	base	Yellow	1663 ^a^	43 ^fg^	n.d. ^h^	131 ^de^	3371 ^a^	4167 ^cde^
	tip	Cream	923 ^bcd^	n.d. ^g^	n.d. ^h^	55 ^e^	1578 ^a^	3347 ^defgh^
Beedance W.	entirety	White	1707 ^a^	56 ^fg^	n.d. ^h^	69 ^e^	1609 ^a^	2157 ^fghi^
9163	entirety	White	1064 ^b^	101 ^ef^	n.d. ^h^	159 ^de^	3273 ^a^	1543 ^hi^

## Data Availability

The data presented in this study are available in the article and the Supplementary Material.

## Supplementary Materials

The following supporting information can be downloaded at: https://www.mdpi.com/article/10.3390/plants11101289/s1, Supplementary Table S1. Content of anthochlors and flavonoids, quantified as aglycones after acidic hydrolysis, in *B. ferulifolia* cultivars. Supplementary Table S2. Selected enzyme activities of the flavonoid and anthochlor pathways detected from enzyme preparations of *B. ferulifolia* cultivars. Supplementary Table S3. Mass spectrometric data of substrates and enzymatically derived products after incubation of isoliquiritigenin or butein in the presence of enzyme preparations of *B. ferulifolia* and NADPH. Supplementary Table S4. Carotenoid yields (mg/g) in extracts of flowers of cultivars Taka Tuka and Bidens gelb (*n* = 3, SD). Supplementary Table S5. Identified compounds by HR-APCI-MS in *B. ferulifolia* cultivars Taka Tuka (T) and Bidens gelb (G). Supplementary Figure S1. Photos showing the undersides of cv. Giant Yellow, cv. Mega Charm and cv. Taka Tuka with and without alkaline vapor treatment. Supplementary Figure S2. Chemical HPLC chromatograms of the methanolic extracts of petal tips of *B. ferulifolia* cv. Bidens gelb and cv. Taka Tuka. Supplementary Figure S3. Chemical structures of carotenoids identified in *B. ferulifolia*.

## References

[1] Crawford D.J., Mort M.E. (2005). Phylogeny of Eastern North American *Coreopsis* (Asteraceae-Coreopsideae): Insights from nuclear and plastid sequences, and comments on character evolution. Am. J. Botany.

[2] Kim K.-J., Choi K.-S., Jansen R.K. (2005). Two chloroplast DNA inversions originated simultaneously during the early evolution of the sunflower family (Asteraceae). Mol. Biol. Evol..

[3] Crowe D.R., Parker W.H. (1981). Hybridization and agamospermy of *Bidens* in northwestern Ontario. Taxon.

[4] Dwyer M. Bidens are biding their time no longer. https://rotarybotanicalgardens.org/bidens-are-biding-their-time-no-longer/.

[5] García-Mendoza A., del Castillo J.M. (2011). Diversidad Florística de Oaxaca: De Musgos a Angispermas.

[6] First registered non-yellow Bidens available in Europe. http://www.newplantsandflowers.com/first-registered-non-yellow-bidens-available-in-europe/.

[7] Brouillard R., Dangles O. (1994). Flavonoids and flower colour. The Flavonoids—Advances in Research.

[8] Scogin R., Zakar K. (1976). Anthochlor pigments and floral UV patterns in the genus *Bidens*. Biochem. Syst. Ecol..

[9] Harborne J.B., Smith D.M. (1978). Anthochlors and other flavonoids as honey guides in the Compositae. Biochem. Syst. Ecol..

[10] Miosic S., Knop K., Hölscher D., Greiner J., Gosch C., Thill J., Kai M., Shrestha B.K., Schneider B., Crecelius A.C. (2013). 4-Deoxyaurone formation in *Bidens ferulifolia* (Jacq.) DC. PLoS ONE.

[11] McCrea K.D., Levy M. (1983). Photographic visualization of floral colors as perceived by honeybee pollinators. Am. J. Bot..

[12] Briscoe A.D., Chittka L. (2001). The Evolution of Color Vision in Insects. Annu. Rev. Entomol..

[13] Harborne J.B. (1967). Comparative Biochemistry of Flavonoids.

[14] Bomati A.K., Austin M.B., Bowman M.E., Dixon R.A., Noel J.P. (2005). Structural elucidation of chalcone reductase and implications for deoxychalcone biosynthesis. J. Biol. Chem..

[15] Halbwirth H., Muster G., Stich K. (2008). Unraveling the biochemical base of dahlia flower coloration. Nat. Prod. Commun..

[16] Bohm B.A. (1988). The minor flavonoids. The Flavonoids: Advances in Research Since 1980.

[17] Bohm B.A. (1993). The minor flavonoids. The Flavonoids: Advances in Research Since 1986.

[18] Boucherle B., Peuchmaur M., Boumendjel A., Haudecoeur R. (2017). Occurrences, biosynthesis and properties of aurones as high-end evolutionary products. Phytochemistry.

[19] Nakayama T., Sato T., Fukui Y., Yonekura-Sakakibara K., Hayashi K., Tanaka Y., Kusumi T., Nishino T. (2000). Aureusidin synthase: A polyphenol oxidase homolog responsible for flower coloration. Science.

[20] Nakayama T., Sato M., Fukui Y., Yonekura-Sakakibara K., Hayashi H., Tanaka Y., Kusumi T., Nishino T. (2001). Specifity analysis and mechanism of aurone synthasis catalyzed by aureusidin synthase, a polyphenol oxidase homolog responsible for flower coloration. FEBS Lett..

[21] Sato T., Nakayama T., Kikuchi S., Fukui Y., Yonekura-Sakakibara K., Ueda T., Nishino T., Tanaka Y., Kusumi T. (2001). Enzymatic formation of aurones in the extracts of yellow snapdragon flowers. Plant Sci..

[22] Ono E., Hatayama M., Isono Y., Sato T., Watanabe R., Yonekura-Sakakibara K., Fukuchi-Mizutani M., Tanaka Y., Kusumi T., Nishino T. (2006). Localization of a flavonoid biosynthetic polyphenol oxidase in vacuoles. Plant J..

[23] Ono E., Fukuchi-Mizutani M., Nakamura N., Fukui Y., Yonekura-Sakakibara K., Yamaguchi M., Nakayama T., Tanaka T., Kusumi T., Tanaka Y. (2006). Yellow flower generated by expression of the aurone biosynthetic pathway. Proc. Natl. Acad. Sci. USA.

[24] Molitor C., Mauracher S.G., Pargan S., Mayer R.L., Halbwirth H., Rompel A. (2015). Latent and active aurone synthase from petals of C. grandiflora: A polyphenol oxidase with unique characteristics. Planta.

[25] Kaintz C., Molitor C., Thill J., Kampatsikas I., Michael C., Halbwirth H., Rompel A. (2014). Cloning and functional expression in *E. coli* of a polyphenol oxidase transcript from *C. grandiflora* involved in aurone formation. FEBS Lett..

[26] Schlangen K., Miosic S., Thill J., Halbwirth H. (2010). Cloning, functional expression, and characterization of a chalcone-3-hydroxylase from *Cosmos sulphureus*. J. Exp. Bot..

[27] Schlangen K., Miosic S., Halbwirth H. (2010). Allelic variants from *Dahlia variabilis* encode flavonoid 3′-hydroxylases with functional differences in chalcone 3-hydroxylase activity. Arch. Biochem. Biophys..

[28] Molitor C., Mauracher S.G., Rompel A. (2016). Aurone synthase is a catechol oxidase with hydroxylase activity and provides insights into the mechanism of plant polyphenol oxidases. Proc. Natl. Acad. Sci. USA.

[29] Zhao D., Tao J. (2015). Recent advances on the development and regulation of flower color in ornamental plants. Front. Plant Sci..

[30] Zhao D., Hao Z., Tao J. (2012). Effects of shade on plant growth and flower quality in the herbaceous peony (*Paeonia lactiflora* Pall.). Plant Physiol. Biochem..

[31] Weiss D. (2000). Regulation of flower pigmentation and growth: Multiple signaling pathways control anthocyanin synthesis in expanding petals. Physiol. Plant..

[32] Walliser B., Lucaciu C.R., Molitor C., Marinovic S., Nitarska D.A., Aktaş D., Rattei T., Kampatsikas I., Stich K., Haselmair-Gosch C. (2021). *Dahlia variabilis* cultivar ‘Seattle’as a model plant for anthochlor biosynthesis. Plant Physiol. Biochem..

[33] Berim A., Gang D.R. (2013). The roles of a flavone-6-hydroxylase and 7-O-demethylation in the flavone biosynthetic network of sweet basil. J. Biol. Chem..

[34] Halbwirth H., Stich K. (2006). An NADPH and FAD dependent enzyme catalyzes hydroxylation of flavonoids in position 8. Phytochemistry.

[35] Ohmiya A. (2011). Diversity of carotenoid composition in flower petals. Jpn. Agric. Res. Q.: JARQ.

[36] Valadon L., Mummery R.S. (1971). Carotenoids of Compositae flowers. Phytochemistry.

[37] Rodriguez-Amaya D.B., Kimura M. (2004). HarvestPlus Handbook for Carotenoid Analysis.

[38] Rodrigues D.B., Mercadante A.Z., Mariutti L.R.B. (2019). Marigold carotenoids: Much more than lutein esters. Food Res. Int..

[39] Haselmair-Gosch C., Miosic S., Nitarska D., Walliser B., Roth B.L., Paltram R., Lucaciu R.C., Eidenberger L., Rattei T., Olbricht K. (2018). Great Cause—Small Effect: Undeclared genetically engineered orange petunias harbor an inefficient dihydroflavonol 4-reductase. Front. Plant Sci..

[40] Thill J., Miosic S., Ahmed R., Schlangen K., Muster G., Stich K., Halbwirth H. (2012). ‘Le Rouge et le Noir’: A decline in flavone formation correlates with the rare color of black dahlia (*Dahlia variabilis* hort.) flowers. BMC Plant Biol..

[41] Doppler P., Kornpointner C., Halbwirth H., Remias D., Spadiut O. (2021). Tetraedron minimum, First reported member of Hydrodictyaceae to accumulate secondary carotenoids. Life.

[42] Petry F.C., Mercadante A.Z. (2018). New method for carotenoid extraction and analysis by HPLC-DAD-MS/MS in freeze-dried citrus and mango pulps. J. Braz. Chem. Soc..

[43] Petry F.C., Mercadante A.Z. (2016). Composition by LC-MS/MS of new carotenoid esters in mango and citrus. J. Agric. Food Chem..

[44] Mercadante A.Z., Rodrigues D.B., Petry F.C., Mariutti L.R.B. (2017). Carotenoid esters in foods—A review and practical directions on analysis and occurrence. Food Res. Int..

[45] Bonaccorsi I., Cacciola F., Utczas M., Inferrera V., Giuffrida D., Donato P., Dugo P., Mondello L. (2016). Characterization of the pigment fraction in sweet bell peppers (*Capsicum annuum* L.) harvested at green and overripe yellow and red stages by offline multidimensional convergence chromatography/liquid chromatography–mass spectrometry. J. Sep. Sci..

[46] De Mendiburu F. (2014). Agricolae: Statistical procedures for agricultural research. R Package Version.

[47] Duncan D.B. (1955). Multiple range and multiple F tests. Biometrics.

