# Supplementary material for: The (Bio)chemical Base of Flower Colour in Bidens ferulifolia
Source: Plants (Basel). 2022 May 11;11(10):1289. doi: 10.3390/plants11101289 (PMC9145775; doi:10.3390/plants11101289)
Supplement: Supplementary file 1 [file plants-11-01289-s001.zip › plants-1705886-supplementary.pdf]

Supplementary Table S1. Content of anthochlors and flavonoids, quantified as aglycones after acidic hydrolysis, in *B. ferulifolia* cultivars. Subtotals for anthochlors and each flavonoid class are shown. The column 'Total' includes anthochlors, flavones, anthocyanins and dihydroflavonols. All measurements are given in µg/g fresh weight. Same letters (a-l) indicate no statistically significant differences according to Duncan ($p<0.05$) between different varieties, n.d.: not detected.

Cv/Line	Petal part	Colour		Anthochlors				Flavones			Anthocyanins			Dihydroflavonols		
				Butein	Okanin	Maritimetin	∑	Apigenin	Luteolin	∑	Cyanidin	Peonidin	∑	DHK	DHQ	∑
9157	entirety	Purple	µg/g	n.d.^{h}	n.d.^{j}	n.d.^{i}	n.d.^{l}	266^{b}	2617^{a}	2944^{b}	345^{d}	n.d.^{b}	345^{d}	31^{a}	1222^{a}	1261
			σ					61	127	188	82		82	8	146	141
Firewheel	base	Red	µg/g	248^{cdefg}	5921^{def}	15^{fgh}	6184^{efgh}	n.d.^{c}	787^{ef}	787^{fg}	246^{e}	3^{a}	249^{e}	n.d.^{b}	148^{e}	148
			σ	17	551	7	539		30	30	14	1	15		5	5
	tip	Red	µg/g	227^{cdefgh}	5026^{efg}	19^{efgh}	5272^{ghi}	n.d.^{c}	1090^{d}	1090^{e}	1806^{a}	n.d.^{b}	1806^{a}	n.d.^{b}	480^{b}	480
			σ	34	268	4	231		148	148	105		105		77	77
Painted Red	base	Red-Yellow	µg/g	204^{defgh}	9019^{abc}	n.d.^{i}	9223^{abcd}	n.d.^{c}	594^{ghi}	594^{hi}	19^{f}	n.d.^{b}	19^{f}	n.d.^{b}	n.d.^{f}	n.d.
			σ	11	293		304		41	41	5		5			
	tip	Red	µg/g	222^{cdefgh}	8087^{abcd}	24^{efgh}	8334^{bcde}	n.d.^{c}	559^{hi}	559^{hi}	1597^{b}	n.d.^{b}	1597^{b}	n.d.^{b}	n.d.^{f}	n.d.
			σ	69	595	3	531		121	121	71		71			
Bidens gelb	entirety	Yellow	µg/g	191^{defgh}	1787^{ij}	9^{hi}	1987^{kl}	n.d.^{c}	152^{l}	152^{lm}	n.d.^{f}	n.d.^{b}	n.d.^{f}	n.d.^{b}	n.d.^{f}	n.d.
			σ	19	24	4	26		21	21						
3176	base	Yellow	µg/g	187^{defgh}	3359^{ghi}	10^{ghi}	3556^{ijk}	n.d.^{c}	338^{jk}	338^{jk}	n.d.^{f}	n.d.^{b}	n.d.^{f}	n.d.^{b}	n.d.^{f}	n.d.
			σ	46	273	5	259		18	18						
	tip	Red	µg/g	791^{a}	10249^{a}	56^{bc}	11095^{a}	n.d.^{c}	1287^{c}	1287^{d}	n.d.^{f}	n.d.^{b}	n.d.^{f}	n.d.^{b}	n.d.^{f}	n.d.
			σ	56	271	17	200		157	157						
3277 (Star type)	edge	Yellow	µg/g	197^{efgh}	6046^{ij}	26^{defgh}	6269^{efgh}	n.d.^{c}	193^{kl}	193^{kl}	n.d.^{f}	n.d.^{b}	n.d.^{f}	n.d.^{b}	n.d.^{f}	n.d.
			σ	51	590	12	625		9	12						
	star	Red	µg/g	636^{cd}	7652^{ab}	27^{defg}	8315^{bcde}	n.d.^{c}	794^{ef}	794^{fg}	n.d.^{f}	n.d.^{b}	n.d.^{f}	n.d.^{b}	n.d.^{f}	n.d.
			σ	37	102	5	134		157	126						
Blazing Embers	base	Yellow	µg/g	113^{efgh}	7013^{cde}	76^{a}	7202^{cdefg}	n.d.^{c}	685^{fgh}	685^{gh}	n.d.^{f}	n.d.^{b}	n.d.^{f}	n.d.^{b}	n.d.^{f}	n.d.
			σ	26	1644	27	1646		27	289						
	tip	Red	µg/g	64^{gh}	3129^{ghi}	60^{b}	3253^{ijk}	n.d.^{c}	176^{kl}	176^{klm}	n.d.^{f}	n.d.^{b}	n.d.^{f}	n.d.^{b}	n.d.^{f}	n.d.
			σ	9	94	8	97		19	297						
Painted Yellow	base	Red	µg/g	27^{gh}	1945^{i}	42^{ghi}	2015^{kl}	n.d.^{c}	n.d.^{l}	n.d.^{m}	517^{c}	n.d.^{b}	517^{c}	n.d.^{b}	150^{e}	150
			σ	9	68	12	66				36		36		34	34
	tip	Yellow	µg/g	58^{fgh}	1503^{ij}	16^{efgh}	1577^{kl}	n.d.^{c}	n.d.^{l}	n.d.^{m}	250^{e}	n.d.^{b}	250^{e}	n.d.^{b}	n.d.^{f}	n.d.
			σ	39	218	7	257				16		16			

Table is continued on next page.

Cv/Line	Petal part	Colour		Anthochlors				Flavones			Anthocyanins			Dihydroflavonols		
				Butein	Okanin	Maritimetin	∑	Apigenin	Luteolin	∑	Cyanidin	Peonidin	∑	DHK	DHQ	∑
Giant	base	Yellow	µg/g	253^{cdefg}	7844^{abcd}	13^{gh}	8110^{bcdef}	n.d.^{c}	533^{hi}	533^{hi}	5^{f}	n.d.^{b}	5^{f}	n.d.^{b}	n.d.^{f}	n.d.
			σ	62	982	6	1045		88	88	3		3			
	tip	Yellow	µg/g	133^{defgh}	1440^{ij}	20^{efgh}	1593^{kl}	n.d.^{c}	39^{l}	39^{lm}	28^{f}	n.d.^{b}	28^{f}	n.d.^{b}	n.d.^{f}	n.d.
			σ	28	117	10	139		7	7	6		6			
Mega Charm	base	Yellow	µg/g	229^{cdefg}	6585^{cdef}	15^{ghi}	6829^{defgh}	n.d.^{c}	764^{efg}	764^{fg}	n.d.^{f}	n.d.^{b}	n.d.^{f}	n.d.^{b}	n.d.^{f}	n.d.
			σ	30	136	6	120		69	69						
	tip	Yellow	µg/g	129^{defgh}	1874^{ij}	n.d.^{i}	2003^{kl}	n.d.^{c}	170^{kl}	170^{lm}	n.d.^{f}	n.d.^{b}	n.d.^{f}	n.d.^{b}	n.d.^{f}	n.d.
			σ	9	215	n.d.	215		14	14						
Eldoro Red Nails	base	Yellow	µg/g	79^{fgh}	4421^{efgh}	14^{ghi}	4514^{hij}	n.d.^{c}	458^{ij}	458^{ij}	n.d.^{f}	n.d.^{b}	n.d.^{f}	n.d.^{b}	n.d.^{f}	n.d.
			σ	20	158	9	149		99	99						
	tip	Red	µg/g	211^{cdefgh}	4554^{efgh}	23^{efgh}	4788^{ghij}	n.d.^{c}	421^{ij}	421^{ij}	1627^{b}	n.d.^{b}	1627^{b}	n.d.^{b}	323^{c}	323
			σ	68	684	11	628		64	64	141		141		49	49
Eldoro Yellow Red Star	star	Red	µg/g	358^{cd}	9625^{ab}	17^{fghi}	10000^{ab}	n.d.^{c}	583^{hi}	583^{hi}	353^{d}	n.d.^{b}	353^{d}	n.d.^{b}	n.d.^{f}	n.d.
			σ	93	380	5	365		43	43	30		30			
	edge	Yellow	µg/g	110^{efgh}	1414ij	20^{efgh}	1545^{kl}	n.d.^{c}	46^{l}	46^{lm}	n.d.^{f}	n.d.^{b}	n.d.^{f}	n.d.^{b}	n.d.^{f}	n.d.
			σ	36	88	7	61		13	13						
Taka Tuka	base	Yellow	µg/g	340^{cde}	9112^{abc}	61^{ab}	9513^{abc}	n.d.^{c}	901^{e}	901^{f}	n.d.^{f}	n.d.^{b}	n.d.^{f}	n.d.^{b}	n.d.^{f}	n.d.
			σ	34	504	2	540		715	715						
	tip	Cream	µg/g	277^{cdef}	2233^{hij}	33^{def}	2543^{jkl}	n.d.^{c}	112^{l}	112^{lm}	n.d.^{f}	n.d.^{b}	n.d.^{f}	n.d.^{b}	n.d.^{f}	n.d.
			σ	29	298	35	362		105	105						
3267	base	Yellow	µg/g	441^{bc}	8815^{abc}	35^{de}	9291^{abcd}	n.d.^{c}	1371^{bc}	1371^{d}	n.d.^{f}	n.d.^{b}	n.d.^{f}	n.d.^{b}	n.d.^{f}	n.d.
			σ	491	5385	9	5885		102	102						
	tip	Cream	µg/g	754^{a}	4855^{efg}	18^{efghi}	5627^{fghi}	n.d.^{c}	416^{ij}	416^{ij}	n.d.^{f}	n.d.^{b}	n.d.^{f}	n.d.^{b}	n.d.^{f}	n.d.
			σ	52	288	15	355		45	45						
Beedance White	entirety	White	µg/g	n.d.^{h}	n.d.^{j}	n.d.^{i}	n.d.^{l}	439^{a}	2754^{a}	3252^{a}	7^{f}	n.d.^{b}	7^{f}	n.d.^{b}	221^{d}	221
			σ					59	306	249	4		4		23	23
9163	entirety	White	µg/g	n.d.^{h}	n.d.^{j}	n.d.^{i}	n.d.^{l}	285^{b}	1498^{b}	1806^{c}	n.d.^{f}	n.d.^{b}	n.d.^{f}	n.d.^{b}	47^{f}	47
			σ					23	197	174					8	8

Supplementary Table S2. Selected enzyme activities of the flavonoid and anthochlor pathways detected from enzyme preparations of *B. ferulifolia* cultivars. Same letters (a-i) indicate no statistical significant differences according to Duncan ($p<0.05$) between different varieties, n.d.: not detected.

**Cv/Line**	**Petal section**	**Colour**	**CHS**	**FHT**	**DFR**	**FNSII**	**CH3H**	**CH3'H**
					Specific activity [nmols^{-1}kg^{-1}]			
9157	entirety	Purple	852^{bcd} ± 164	321^{b} ± 84	1286^{a} ± 226	183^{de} ± 17	2121^{a} ± 314	1642^{ghi} ± 478
Firewheel	base	Red	383^{ghi} ± 62	90^{ef} ± 9	473^{cd} ± 39	417^{ab} ± 62	3663^{a} ± 827	7472^{a} ±1511
	tip	Red	253^{i} ± 26	145^{de} ± 9	892^{b} ± 82	469^{a} ± 90	4463^{a} ± 1626	3367^{defgh} ± 313
Painted Red	base	Red-Yellow	774^{cde} ± 142	n.d.^{g}	605^{c} ± 68	435^{a} ± 194	3568^{a} ± 3568	4701 ^{bcd} ±4701
	tip	Red	658^{cdefg} ± 23	546^{a} ± 85	975^{b} ± 176	391^{abc} ± 42	3704^{a} ± 198	5989^{ab} ± 661
Bidens gelb	entirety	Yellow	465^{fghi} ± 146	69^{fg} ± 12	n.d.^{h}	162^{de} ± 40	2988^{a} ± 1304	3414^{defgh} ± 971
3176	base	Yellow	219^{i} ± 74	219^{c} ± 35	11^{h} ± 14	400^{abc} ± 267	3519^{a} ± 2919	4000^{cdef} ± 872
	tip	Red	250^{i} ± 11	249^{c} ± 18	188^{fg} ± 49	418^{ab} ± 101	5589^{a} ± 3125	3980^{cdef} ± 138
3277	edge	Yellow	489^{efghi} ± 63	n.d.^{g}	55^{gh} ± 44	383^{abc} ± 175	4386^{a} ± 4386	4762 ^{bcd} ±4762
	star	Red	734^{cdef} ± 273	381^{b} ± 77	240^{ef} ± 87	315^{abcd} ± 94	3781^{a} ± 711	5619^{bc} ±1569
Blazing Embers	base	Yellow	226^{i} ± 70	n.d.^{g}	20^{g} ± 19	118^{de} ± 33	2310^{a} ± 1076	3159 ^{defghi} ±3159
	tip	Red	276^{hi} ± 67	242^{c} ± 31	153^{fgh} ± 30	179^{de} ± 25	1996^{a} ± 555	2184^{fghi} ± 346
Painted Yellow	base	Red	446^{fghi} ± 126	180^{cd} ± 38	377^{de} ± 41	231^{bcde} ± 24	2619^{a} ± 913	2454^{efghi} ± 702
	tip	Yellow	483^{cde} ± 179	n.d.^{g}	18^{h} ± 23	131^{de} ± 14	2870^{a} ± 1395	3101 ^{cdef} ±3101
Giant	base	Yellow	480^{fghi} ± 63	n.d.^{g}	255^{ef} ± 85	172^{de} ± 44	3640^{a} ± 3019	3958 ^{cdef} ±3958
	tip	Yellow	403^{ghi} ± 25	n.d.^{g}	559^{c} ± 70	104^{e} ± 9	1769^{a} ± 268	3505 ^{defgh} ±3505
Mega Charm	base	Yellow	430^{ghi} ± 160	n.d.^{g}	n.d.^{h}	46^{e} ± 9	2795^{a} ± 2745	4135 ^{cde} ±4135
	tip	Yellow	217^{i} ± 54	n.d.^{g}	n.d.^{h}	70^{e} ± 15	1730^{a} ± 1370	1366^{i} ±1366
Eloro. Red Nails	base	Yellow	412^{ghi} ± 79	n.d.^{g}	n.d.^{h}	218^{cde} ± 68	3880^{a} ± 955	3126 ^{defghi} ±3126
	tip	Red	436^{ghi} ± 78	375^{b} ± 26	1028^{b} ± 74	221^{cde} ± 35	3617^{a} ± 327	3400^{defgh} ± 321
Eldoro Yellow Red Star	star	Red	561^{efghi} ± 19	187^{cd} ± 10	435^{d} ± 54	184^{de} ± 18	2878^{a} ± 711	2437^{efghi} ± 433
	edge	Yellow	490^{defgh} ± 131	n.d.^{g}	n.d.^{h}	45^{e} ± 3	1363^{a} ± 1091	1751^{ghi} ± 211
Taka Tuka	base	Yellow	827^{bcd} ± 51	30^{fg} ± 7	n.d.^{h}	137^{de} ± 23	2683^{a} ± 1523	2736 ^{efghi} ±2736
	tip	Cream	786^{bcd} ± 51	n.d.^{g}	n.d.^{h}	60^{e} ± 12	1732^{a} ± 1497	3376^{defgh} ± 856
3267	base	Yellow	1663^{a} ± 54	43^{fg} ± 10	n.d.^{h}	131^{de} ± 28	3371^{a} ± 2808	4167 ^{cde} ±4167
	tip	Cream	923^{bcd} ± 85	n.d.^{g}	n.d.^{h}	55^{e} ± 14	1578^{a} ± 891	3347^{defgh} ± 423
Beedance White	entirety	White	1707^{a} ± 361	56^{fg} ± 14	n.d.^{h}	69^{e} ± 19	1609^{a} ± 1126	2157 ^{fghi} ±2157
9163	entirety	White	1064^{b} ± 110	101^{ef} ± 14	n.d.^{h}	159^{de} ± 28	3273^{a} ± 775	1543 ^{hi} ±1543

Supplementary Table S3. Mass spectrometric data of substrates and enzymatically derived products after incubation of isoliquiritigenin or butein in the presence of enzyme preparations of *B. ferulifolia* and NADPH.

Analytes	Retention time	λmax (nm)	Theoretical precursor ion $[M-H]^-$ (m/z)	Measured precursor ion $[M-H]^-$ (m/z)	Δppm	Fragment ions (m/z)
Isoliquiritigenin	11.98	239.8; 368.5	255.0663	255.0664	0,3921	119.0501; 135.0085
3'-Hydroxyisoliquiritigenin	10.69	238.8; 366.3	271.0612	271.0610	-0.7378	119.0500; 151.0033
Butein	11.01	261.1; 381.3	271.0612	271.0605	-2.5824	135.0452; 135.0084
Okanin	9.80	259.8; 378.6	287.0561	287.0557	-1.3935	135.0450: 151.0036

Supplementary Table S4. Carotenoid yields (mg/g) in extracts of flowers of cultivars Taka Tuka and Bidens gelb (n = 3, SD). Same letters (a-c) indicate no statistical differences in the same column according to Duncan ($p<0.05$), n.d.: not detected, Xanthophylls and carotenoid esters are expressed in (all-*E*)-lutein equivalents

Cv/line	Petal section	(all-*E*)-lutein (mg/g)	Total free carotenoids (mg/g)	Total monoesters (mg/g)	Total diesters (mg/g)	Total carotenoids (mg/g)
Taka Tuka	base	0.37^{b} ± 0.04	0.59^{a} ± 0.04	0.062^{c} ± 0.0014	n.d.^{c}	0.65^{c} ± 0.04
	tip	0.50^{a} ± 0.07	0.67^{a} ± 0.08	0.032^{c} ± 0.008	n.d.^{c}	0.70^{bc} ± 0.09
Bidens gelb	base	0.112^{c} ± 0.013	0.184^{c} ± 0.021	0.54^{b} ± 0.05	0.087^{a} ± 0.028	0.81^{b} ± 0.08
	tip	0.172^{c} ± 0.004	0.335^{b} ± 0.006	1.29^{a} ± 0.04	0.066^{b} ± 0.011	1.69^{a} ± 0.05

Supplementary Table S5. Identified compounds by UHPLC-DAD-APCI-MS in *B. ferulifolia* cultivars Taka Tuka (T) and Bidens gelb (G). The corresponding chromatograms are shown in Suppl. Figure S3.

Peak (sample)	Compound	λ_{max} (nm)	$[M+H]^+$ (*m/z*)	Fragment ions (*m/z*)
1 (G)	unidentified	416; 439; 468	-	-
2 (G, T)	(13*Z*)-violaxanthin	416, 440, 468	601.4	583.4 $[M+H-H_2O]^+$; 565.4 $[M+H-2H_2O]^+$
3 (G, T)	(all-*E*)-violaxanthin	415, 437, 467	601.4	583.4 $[M+H-H_2O]^+$; 565.4 $[M+H-2H_2O]^+$; 509.4 $[M+H-92]^+$; 491.4$[M+H-H_2O-92]^+$
4a (G, T)	(all-*E*)-luteoxanhin	399, 422, 448	601.4	583.4 $[M+H-H_2O]^+$; 565.4 $[M+H-2H_2O]^+$; 509.4 $[M+H-92]^+$; 491.4$[M+H-H_2O-92]^+$
4b (G, T)	(9*Z*)-violaxanthin	415, 440, 469	601.4	583.4 $[M+H-H_2O]^+$; 565.4 $[M+H-2H_2O]^+$
5 (G, T)	lutein 5,6-epoxide	415, 437, 466	585.4	567.4 $[M+H-H_2O]^+$; 549.4 $[M+H-2H_2O]^+$
6 (G, T)	(all-*E*)-antheraxanthin	Sh420, 442, 469	585.4	567.4 $[M+H-H_2O]^+$; 549.4 $[M+H-2H_2O]^+$
7 (T)	(9*Z*)-luteoxanthin	397, 420, 446	601.4	583.4 $[M+H-H_2O]^+$; 565.4 $[M+H-2H_2O]^+$
8 (G)	unidentified	416, 438, 467	-	549.4 $[M+H-2H_2O]^+$
9 (G)	(13*Z*)-lutein	414, 437, 466	569.4	551.4 $[M+H-H_2O]^+$; 533.4 $[M+H-2H_2O]^+$; 459.4 $[M+H-H_2O-92]^+$
10 (G, T)[a]	(all-*E*)-lutein	Sh420, 442, 470	569.4	551.4 $[M+H-H_2O]^+$; 533.4 $[M+H-2H_2O]^+$; 459.4 $[M+H-H_2O-92]^+$
11 (G, T)	(all-*E*)-zeaxanthin	Sh420, 446, 470	569.4	551.4 $[M+H-H_2O]^+$; 533.4 $[M+H-2H_2O]^+$; 477.4 $[M+H-92]^+$; 459.4 $[M+H-H_2O-92]^+$
12 (T)	(9*Z*)-lutein	Sh415, 437, 465	569.4	551.4 $[M+H-H_2O]^+$; 533.4 $[M+H-2H_2O]^+$
13 (T, G)	chlorophyll b	465, 650	-	-
14 (T, G)	chlorophyll a	430, 664	-	-
15 (G)	(all-*E*)-violaxanthin myristate	417, 439, 468	811.6	793.6 $[M+H-H_2O]^+$; 775.7 $[M+H-2H_2O]^+$; 701.6 $[M+H-H_2O-92]^+$; 565.4 $[M+H-H_2O-My]^+$
16 (G)	(9*Z*)-violxanthin myristate	412, 435, 463	811.6	793.6 $[M+H-H_2O]^+$; 565.4 $[M+H-H_2O-My]^+$
17a (G)	violaxanthin palmitate	416, 439, 468	839.7	821.6 $[M+H-H_2O]^+$; 803.6 $[M+H-2H_2O]^+$; 729.6 $[M+H-H_2O-92]^+$; 565.4 $[M+H-H_2O-Pa]^+$
17b (G)	violaxanthin palmitate	414, 438, 467	839.7	821.6 $[M+H-H_2O]^+$; 803.6 $[M+H-2H_2O]^+$; 729.6 $[M+H-H_2O-92]^+$; 565.4 $[M+H-H_2O-Pa]^+$
18 (G)	(all-*E*)-luteoxanthin palmitate	Sh401, 420, 446	839.7	821.6 $[M+H-H_2O]^+$; 565.4 $[M+H-H_2O-Pa]^+$
19 (G)	(9*Z*)-violaxanthin palmitate	413, 435, 463	839.7	821.6 $[M+H-H_2O]^+$; 565.4 $[M+H-H_2O-Pa]^+$
20 (G)	lutein 5,6-epoxide palmitate	414, 439, 468	823.6	805.7 $[M+H-H_2O]^+$; 549.4 $[M+H--H_2O-Pa]^+$
21 (G)	(13*Z*)-lutein myristate	415, 438, 467	779.6	761.6 $[M+H-H_2O]^+$; 551.4 $[M+H-My]^+$; 533.4 $[M+H-My-H_2O]^+$; 495.4 $[M+H-My-56]^+$ 459.4 $[M+H-My-92]^+$
22 (G, T)	(all-*E*)-lutein 3′-*O*-myristate	Sh421, 444, 471	779.6	551.4 $[M+H-My]^+$; 533.4 $[M+H-My-H_2O]^+$; 459.4 $[M+H-My-92]^+$
23 (G, T)	(all-*E*)-lutein 3-*O*-myristate	Sh420, 443, 470	779.6	761.6 $[M+H-H_2O]^+$; 551.4 $[M+H-My]^+$; 533.4 $[M+H-My-H_2O]^+$
24 (T)[a]	(all-*E*)-β-carotene	Sh425, 449, 474	537.4	536.4 $[M]^+$; 445.4 $[M+H-92]^+$
25a (G)	(all-*E*)-zeaxanthin myristate	Sh420, 442, 468	779.6	761.6 $[M+H-H_2O]^+$; 551.4 $[M+H-My]^+$; 533.4 $[M+H-My-H_2O]^+$
25b (G)	unidentified	416, 440, 468	-	-

Table is continued on next page.

Peak (sample)	Compound	λ_{max} (nm)	$[M+H]^+$ (*m*/*z*)	Fragment ions (*m*/*z*)
26 (G)	(13*Z*)-lutein palmitate	415, 438, 467	807.7	789.7 $[M+H-H_2O]^+$; 551.4 $[M+H-Pa]^+$; 533.4 $[M+H-Pa-H_2O]^+$
27 (G, T)	(all-*E*)-lutein 3′-*O*-palmitate	Sh421, 444, 471	807.7	551.4 $[M+H-Pa]^+$; 533.4 $[M+H-Pa-H_2O]^+$
28 (G, T)	(all-*E*)-lutein 3-*O*-palmitate	Sh420, 444, 471	807.7	789.7 $[M+H-H_2O]^+$; 697.6 $[M+H-H_2O-92]^+$; 551.4 $[M+H-Pa]^+$; 533.4 $[M+H-Pa-H_2O]^+$
29 (G, T)	(all-*E*) zeaxanthin palmitate	Sh423, 449, 475	807.7	789.7 $[M+H-H_2O]^+$; 551.4 $[M+H-Pa]^+$; 533.4 $[M+H-Pa-H_2O]^+$
30 (G)	(13*Z*)-lutein stearate	415, 438, 468	835.7	817.7 $[M+H-H_2O]^+$; 533.4 $[M+H-St-H_2O]^+$
31 (G)	(all-*E*)-lutein 3′-*O*-stearate	420, 443,.471	835.7	817.7 $[M+H-H_2O]^+$; 551.4 $[M+H-St]^+$; 533.4 $[M+H-St-H_2O]^+$
32 (G)	(all-*E*)-lutein 3-*O*-stearate	421, 443, 472	835.7	817.7 $[M+H-H_2O]^+$; 551.5 $[M+H-St]^+$; 533.4 $[M+H-St-H_2O]^+$
33 (G)	violaxanthin myristate palmitate	417, 440, 469	1049.9	1031.8 $[M+H-H_2O]^+$; 939.8 $[M+H-92-H_2O]^+$; 547.4 $[M+H-My-Pa]^+$
34 (G)	violaxanthin dipalmitate	416, 439, 468	1077.9	1059.9 $[M+H-H_2O]^+$; 985.8 $[M+H-92]^+$; 803.6 $[M+H-Pa-H_2O]^+$; 547.4 $[M+H-2Pa]^+$
35 (G)	(13*Z*)-lutein dimyristate	416, 439, 468	989.9	761.6 $[M+H-My]^+$; 533.4 $[M+H-2My]^+$
36 (G)	(all-*E*)-lutein dimyristate	421, 444, 471	-	761.6 $[M+H-My]^+$; 669.6 $[M+H-92]^+$; 533.4 $[M+H-2My]^+$
37 (G)	(13*Z*)-lutein myristate palmitate	415, 439, 468	-	789.7 $[M+H-My]^+$; 761.6 $[M+H-Pa]^+$; 533.4 $[M+H-My-Pa]^+$
38 (G)	(all-*E*)-lutein myristate palmitate	422, 444, 473	-	789.7 $[M+H-My]^+$; 761.6 $[M+H-Pa]^+$; 533.4 $[M+H-My-Pa]^+$
39 (G)	(13*Z*)-lutein dipalmitate	416, 439, 467	-	789.7 $[M+H-Pa]^+$; 533.4 $[M+H-2Pa]^+$

[a]compared to standard substances

My: myristate; Pa: palmitate; St: stearate

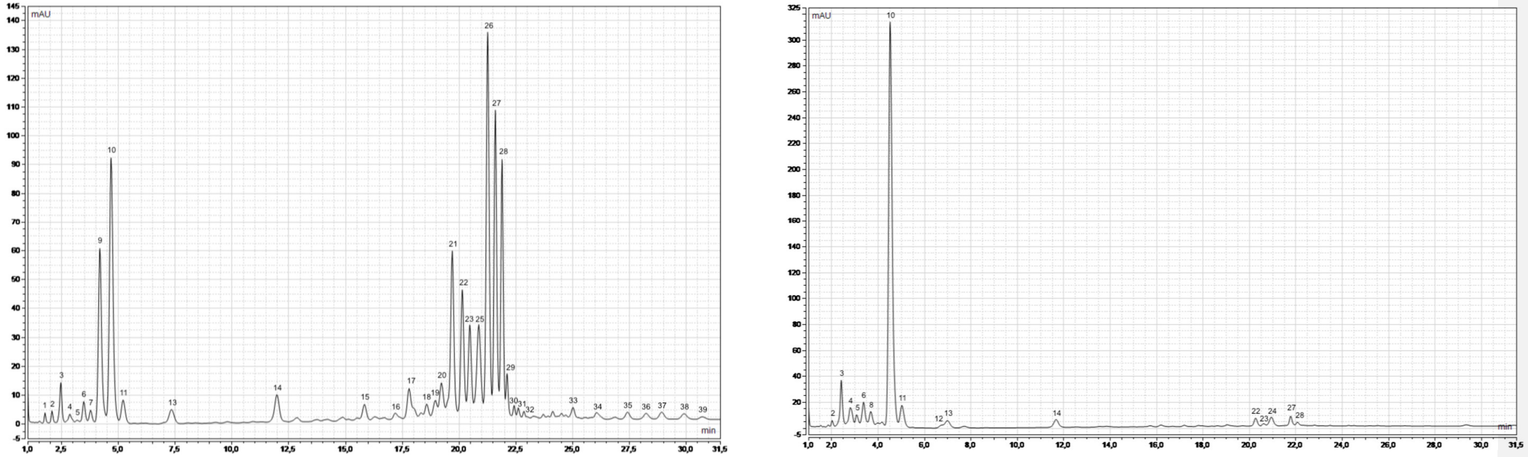

Supplementary Figure S1. HPLC chromatograms (λ = 450 nm) of the methanolic extracts of petal tips of *B. ferulifolia* cv. Bidens gelb (left) and cv. Taka Tuka (right). Peak numbers are resolved in Suppl. Table S4.

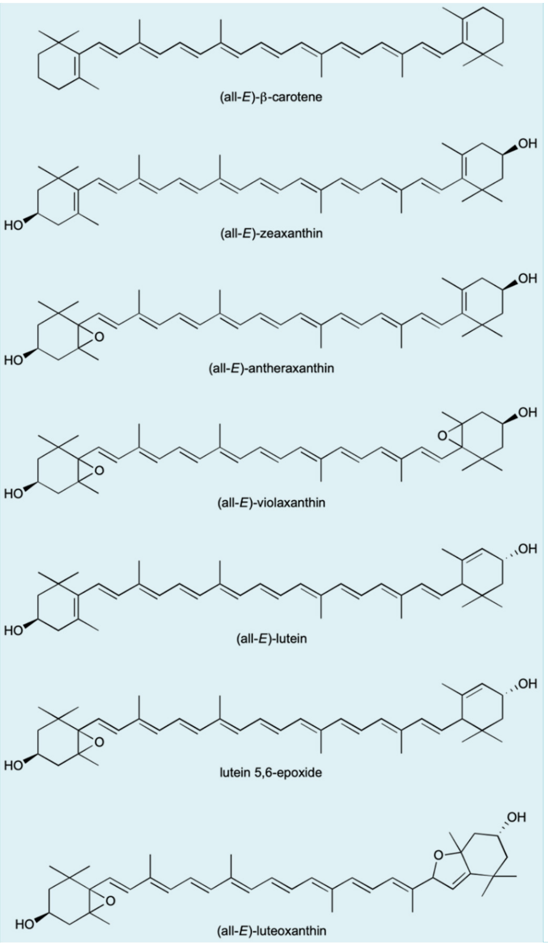

Supplementary Figure S2. Chemical structures of carotenoids identified in *B. ferulifolia*.

Commented [HH1]: was replaced

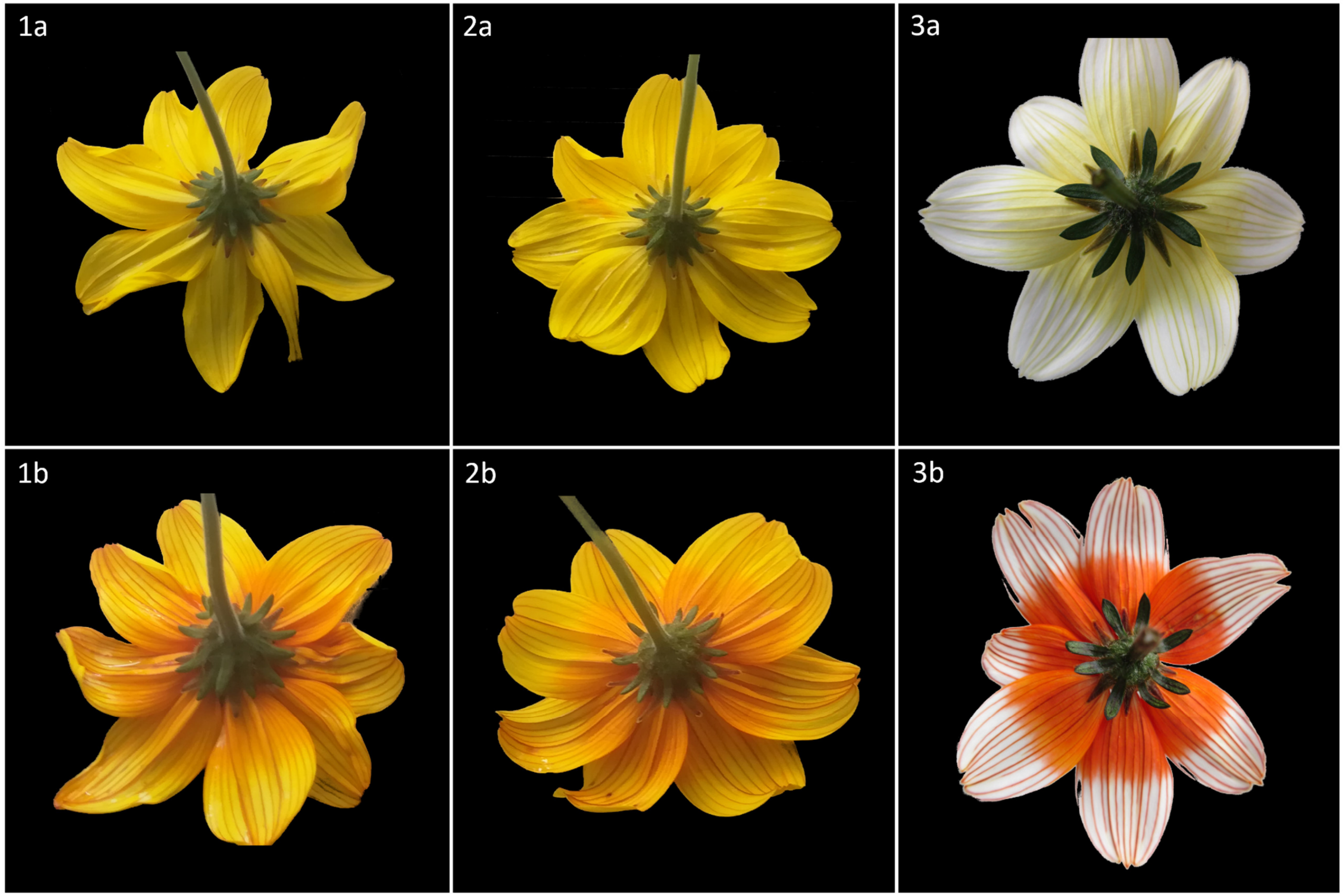

Supplementary Figure S3. Photos showing the undersides of cv. Giant Yellow without (1a) and with (1b) alkaline vapor treatment, cv. Mega Charm without (2a) and with (2b) alkaline vapor treatment, and cv. Taka Tuka without (3a) and with (3b) alkaline treatment.
